# Water, Health, and Environmental Justice in California: Geospatial Analysis of Nitrate Contamination and Thyroid Cancer

**DOI:** 10.1089/ees.2020.0315

**Published:** 2021-05-24

**Authors:** Arianna Q. Tariqi, Colleen C. Naughton

**Affiliations:** Department of Civil and Environmental Engineering, University of California Merced, Merced, California, USA.

**Keywords:** agriculture, disadvantaged communities, groundwater contamination, safe drinking water

## Abstract

Environmental health hazards are known to disproportionately burden marginalized communities. Agriculture, wastewater, and industrial waste contaminate surface and groundwater, used for drinking, with nitrates. High nitrate concentrations in drinking water have been linked to methemoglobinemia and, recently, thyroid cancer. With a large proportion of the nation's agriculture grown in California, thyroid cancer linked to nitrate water contamination is of concern. This research entailed geographic and statistical analysis of water, nitrate, health, and disadvantaged communities (DACs) in California. DACs are Californian defined areas that experience a combination of hardships from socioeconomic, health, and environmental fields. Our analysis of the California Cancer Registry and California Water Board's well data shows statistically significant correlation (*p* < 0.05) between nitrate contamination (wells >5 and 10 ppm NO_3_-N per square mile and percentage of total wells) and thyroid cancer incidence. DACs had twice the rate of thyroid cancer compared with non-DACs, and higher numbers of nitrate-contaminated wells and hot spots compared with the state averages. Almost half (47%) of the Central Valley's area contained DACs and 27% of wells >10 ppm NO_3_-N contaminants. Our study provides a method for other states and countries to conduct preliminary geospatial analysis between water contamination and health with open data. Maps and analysis from this research can inform the public, advocacy groups, and policy leaders of health-related concerns in relation to nitrate water contamination and environmental justice in California. DACs should be provided cost-effective drinking water monitoring and treatment, and governments should incentivize nitrate loading reductions in agriculture, industry, and wastewater. Future research is recommended with more localized, private health data on thyroid cancer incidence.

## Introduction

Nitrates in drinking water have long been studied in relation to the detrimental effects on human health. The natural nitrogen cycle has been disrupted by humans who have more than doubled the nitrogen input into the terrestrial nitrogen cycle since the 1960s (Vitousek *et al.*, 1997). Anthropogenic nitrogen inputs include fertilizers for agriculture, the combustion of fossil fuels, nitrogen-fixing crops (Vitousek *et al.*, 1997; Davidson *et al.*, 2012), lawns, and gardens, improperly disposed household cleaners, industrial and military sources (Wakida and Lerner, 2005) as well as untreated wastewater (Wakida and Lerner, 2005; Harter *et al.*, 2012).

Over 5.6 million Americans drink from community water systems with >5 mg/L or parts per million (ppm) nitrate (the United States Environmental Protection Agency [EPA] standard is 10 ppm) (Schaider *et al.*, 2019). Community water wells in the United States from groundwater sources had four times higher nitrate concentrations than drinking water from surface water sources (Schaider *et al.*, 2019). Since 1945, scientists have found that nitrates in drinking water can cause methemoglobinemia (aka blue baby syndrome), which lead to the creation of the U.S. EPA's drinking water standard of 10 ppm NO_3_-N (Comly, 1987; EPA, 2020a). Similarly, the World Health Organization (WHO)'s Maximum Contaminant Level (MCL) is 11.3 ppm NO_3_-N due to the absence of blue baby syndrome under such levels (WHO, 2017).

Research has emerged on the link between nitrates and thyroid cancer (Ward *et al.*, 2010; Drozd *et al.*, 2016; WHO, 2017). Thyroid cancer is the 9th most common cancer responsible for 3.1% of new cases out of 36 cancers worldwide (Bray *et al.*, 2018). Thyroid cancer represents 5.1% of the female cancer burden (1 in 20 cancer diagnosis) (Bray *et al.*, 2018). Globally, nitrate concentrations in water are expected to rise with population growth, increased application of nitrogen fertilizers with intensified agriculture, and climate change affecting the water cycle with higher nitrogen runoff from intensified rains (Davidson *et al.*, 2012).

Worldwide, fertilizers applied to cropland account for 60% of nitrate contamination in groundwater (Shukla and Saxena, 2018). Nitrate contamination sources and contributions vary locally. Specifically in the Tulare Lake Basin and the Salinas Valley, agricultural areas representing 40% of irrigated cropland in California in or near the Central Valley, fertilizers applied to cropland accounted for 96% of the source for nitrate loading (Harter *et al.*, 2012). Wastewater treatment and food processing waste accounted for 1.5%, leachate from septic systems were 1%, fertilizers applied to urban parks, lawns, and golf courses accounted for <1%, corrals and lagoons were <1%, and nitrate migration downstream was another 1% (Harter *et al.*, 2012).

At the same time, nitrate concentrations are increasing, so is thyroid cancer incidence. The age-standardized world incidence rate is 10.1 per 100,000 in women and 3.1 per 100,000 in men (GCO, 2018). In the United States, thyroid cancer rates were 6.9 and 19.4 per 100,000 people in men and women, respectively, in 2017 (USCS, 2019). Similarly, California's incidence rates of thyroid cancer were 18.8 per 100,000 for women and 6.5 for men (USCS, 2019). In 2015, thyroid cancer was in the top five most prevalent cancers for women in California (Killion *et al.*, 2018).

Thyroid cancer's average annual percent change from 2005 to 2014 was increasing faster than 19 of the other cancers at 4.6 for women and 4.1 for men in cancer incidence (Killion *et al.*, 2018). Horn-Ross *et al.* (2014) concluded that the technological advances in cancer detection starting in the 1980s were not the only reason for the increase in thyroid cancer. Behavioral, lifestyle, or environmental factors (such as endocrine disruptors, which can be found in a variety of everyday items such as plastics) were likely causes for increases in thyroid cancer incidences (Horn-Ross *et al.*, 2014). If these trends continue, thyroid cancer will likely be the fourth most common cancer in the United States by 2030 mostly among adults >65 and minority populations (Rahib *et al.*, 2014).

Nitrates are naturally occurring in many vegetables and legumes, and added to cured meats, such as hot dogs and bacon (Kilfoy *et al.*, 2011). The human body processes nitrates from food differently than nitrates from drinking water. Acids in fresh foods act as antioxidants and reduce the nitrates to nitric acid in the digestive tract. Negative correlations were found between fruit and vegetable intake and cancer in the stomach, larynx, esophagus, mouth, and cervix (Mirvish, 1986). When a person drinks nitrate-contaminated water, the nitrate breaks down to nitrite, nitrosation, and can introduce N-nitroso compounds, which are highly carcinogenic (Ward *et al.*, 2010). Different types of cancers (thyroid, bladder, esophagus, colon, stomach) and other health issues may also be present where there are high nitrate concentrations (Ward *et al.,* 2018; Njeze *et al.*, 2014; Drozd *et al.*, 2016).

Geospatial and statistical analyses of the link between thyroid cancer, thyroid disease, and nitrate concentrations in drinking water have been conducted in several U.S. states. A study conducted in Iowa is believed to be the first study to find positive correlation between thyroid cancer risk and public water systems >5 ppm nitrate over a 5 year period of consumption of water (Ward *et al.*, 2010). The study sample contained >20,000 older women who used their same public water supply and their health data. Another study in the U.S. state of Vermont analyzed thyroid cancer incidence from 1994 to 2007 by zip code (Hanley *et al.*, 2015), and found nonrandom hot spot clusters likely due to socioeconomic and environmental factors (Hanley *et al.*, 2015). In 2012, a study conducted in the Old Order Amish community (3,017 members aged ≥18) in Pennsylvania found an association in women, between an estimated exposure to nitrate at concentrations ≥6.5 ppm and levels of thyroid stimulating hormone (TSH) between 4 and 10 mIU/mL defined as subclinical hypothyroidism (Aschebrook-Kilfoy *et al.*, 2012). Our study combines methodology from these previous studies to fill the missing analysis of California's nitrate contamination in relation to thyroid cancer and vulnerable communities with open data.

California is particularly an important state to investigate the potential relationship between nitrates and thyroid cancer with historical and continued application of nitrogen-based fertilizers. One-third of the United States' vegetables and two-thirds of their fruits and nuts are grown in California (CDFA, 2020). Agriculture is particularly concentrated in the Central Valley of California, which produces one-fourth of the United States' food (USGS, 2020) and 18% of the nation's dairy milk supply (CDFA, 2018). Fertilizers used in agricultural production contain nitrates, which seep from the surface down into groundwater potentially into drinking water supplies.

Dairies are another source of nitrate from their lagoons, corrals, and manure used in fields (Harter *et al.*, 2013). A task report on the Tulare Lake Basin and Salinas Valley in California's San Joaquin Valley also indicated septic tanks as a source for groundwater nitrate contamination (Harter *et al.*, 2013). Rural–suburban septic tank densities (>256 tanks per mile squared) were higher than the EPA recommended 40 septic tanks per square mile (Harter *et al.*, 2013). Groundwater contaminants can persist for many years, cost more to clean up, and disproportionately affect low-income communities (Harter *et al.*, 2012).

The Natural Resources Defense Council (NRDC) indicated 431 counties in the United States that are in the top third for all drinking water violations and top third of racial, ethnic, and language vulnerability (NRDC, 2019). California is one of the top three states with higher numbers of groundwater system violations and number of people served by systems in violation (Pennino *et al.*, 2017). Balazs *et al.*'s (2012) study mentioned a “compliance burden,” which describes the unequal availability for certain communities or groups being able to meet standards set for contaminants. Schaider *et al.* (2019) found that U.S. community water systems (serving ≤10,000 people) with the highest concentrations of nitrates served twice as many Hispanic residents. In the San Joaquin Valley in particular, 95% of the residents rely on the groundwater wells for their drinking water (Balazs *et al.*, 2011). High nitrate concentrations are disproportionately in poorer and predominantly Latino community water systems, particularly in the San Joaquin Valley (Balazs *et al.*, 2011).

Statistics showing lower drinking water quality in Hispanic communities in the United States and California is an environmental justice issue. The EPA states “environmental justice is the fair treatment and meaningful involvement of all people regardless of race, color, national origin, or income, with respect to the development, implementation, and enforcement of environmental laws, regulations, and policies” (EPA, 2020b). To address environmental justice, California Senate Bill (SB) 535 was passed to ensure cap-and-trade money is invested in disadvantaged communities (DACs) (OEHHA, 2018). CalEPA created a tool, CalEnviroScreen, to geospatially determine DACs based on socioeconomic, health, and environmental fields combined (OEHHA, 2018).

Geospatial technology has been increasingly prominent and is powerful in its capabilities to “visualize, analyze, and interpret” health, environmental, and population data (Tim, 1995; Musa *et al.*, 2013; Hanley *et al.*, 2015; Hersh, 2017). Our research goal is to geographically and statistically analyze the potential association between nitrate water contamination and thyroid cancer with focus on the DACs in California most impacted. Researching the connection between thyroid cancer and nitrate contamination in California fills a gap in the literature since an analysis has not been conducted in California and only a few states (Iowa, Vermont, and Pennsylvania). Also we relied on openly available data unlike other studies (Ward *et al.*, 2010; Hanley *et al.*, 2015) to demonstrate how a preliminary analysis can be done with open data.

## Materials and Methods

An overview of data collection and study workflow for the geospatial and statistical analyses is provided in Supplementary Table S1.

### Data sources

Data on nitrate concentrations (mg/L or ppm NO_3_-N) in drinking water wells (domestic, municipal, and water supply all before potential water treatment) were acquired from the California Water Boards (GAMA) website (GAMA, 2020). Dates of these nitrate readings start from 1907 to 2019 ranging from one to multiple readings per well. The entire range was used to maximize the amount of measurements per well for the average concentration over time and since more chronic exposure may be needed to cause thyroid cancer from drinking water with higher levels of nitrates. We downloaded the nitrate well records from the GAMA database for each of the 58 counties in California with location, date, well ID, well type, source, source name, and nitrate concentration in mg/L.

Invasive thyroid cancer incidence data per county were collected from the California Cancer Registry for 2014 (CCR, 2019). Twenty one of California's counties had “unstable” numbers (<15 cases of thyroid cancer). Of these 21 counties, 17 were put into groups of two and three counties according to their location. For our analysis, we replaced the “unstable numbers” with 1, so we could map the low incidence rates of these counties and compare them with the 37 other counties. We divided the total county case data by population per county in 2014 and multiplied by 100,000 to calculate the rate of cancer incidence in each county relative to the county's population (thyroid cases per 100,000). Of the counties that were unstable and combined, population was totaled for the grouped counties.

In addition, a sensitivity analysis was conducted to ensure that county thyroid cancer cases per population did not change drastically with a change in their rates. The analysis was conducted using 15 (the maximum amount of cases), 7.5, 1, and 0. We similarly determined and compared the thyroid cancer case rate per 100,000 for all of California, DACs, Central Valley, the population in California not in DACs, and the population not in the Central Valley.

DAC data were obtained from the California Office of Environmental Health Hazard Assessment (OEHHA) and California Environmental Protection agency (CalEPA) CalEnviroScreen 3.0 geodatabase, last updated June 2018 (OEHHA, 2018). Communities were scored based on their exposure to pollutants (such as PM 2.5, pesticides, and toxic releases from facilities), sensitive populations (including low-birth weights, asthma, and emergency department visits, etc.), environmental effects (e.g., cleanup sites, hazardous waste, etc.), and socioeconomic factors (e.g., poverty, unemployment, or education attainment, etc.). A community is considered disadvantaged if it falls in the top 25% scoring areas (OEHHA, 2018). Latinos and African Americans are more likely to live in communities that are highly impacted by the combined impacts listed in the CalEnviroScreen 3.0 (OEHHA, 2018).

Area and population data were also included and used in our statistical analysis. County shape files were acquired from the California Open Data Portal (California Open Data Portal, 2019). Population and land area by county were obtained from the California State Association of Counties (Counties.org) (CSAC, 2019, 2020). Central Valley data were obtained from the United States Geographical Survey (USGS, 2009).

### Geospatial analysis

ArcGIS Pro version 2.5.1 was used for geospatial analysis to visually represent and analyze the relationship between wells with high nitrate concentrations and their position relative to thyroid cancer incidence, DACs, and the Central Valley. As other studies have done, nitrate concentrations over time were averaged for each well point (Weyer *et al.*, 2001; Ward *et al.*, 2010). The dissolve tool was used to average a multitude of data points from samples with inconsistent sampling date patterns and intervals to display each well as a singular concentration point. Before we started the geospatial analysis, outliers were removed that were outside three standard deviations of the mean (99.7% confidence interval).

Getis-Ord GI* is a spatial statistics tool for calculating hot spots, which requires a distance band for determining our scale of analysis (Deitz and Meehan, 2019). See Supplementary Table S1 for more details on the process. Similar to the Hanley *et al.* (2015) study, this study used the “Calculate Distance band from Neighborhood Count” tool in ArcGIS Pro to find the distance band for the hot spot analysis. Neighborhoods of one and eight were chosen. A maximum neighborhood of one is the largest distance needed for each well to have at least one neighboring well. An average neighborhood of eight is the distance between each well and its' eight closest neighbors averaged throughout California. These distances were the best choice to encompass all of California's well points representative of dense urban areas and spread-out rural areas.

For this study, the distance band encompasses well points and their neighboring wells to find proportionally higher concentrations of nitrates in a neighborhood at a consistent “Fixed Distance,” meaning well points will be viewed only in the context of its neighbors. This limits bias by comparing wells incrementally throughout California, comparing only neighboring ones with one another for statistically significant high nitrate concentration values.

After running the Getis-Ord GI* tool with our distance band and selected analysis method, the hot spot analysis output was categorized into seven intervals starting from statistically significant “cold spots” to “not significant points” and finally “hot spots.” These intervals are determined by statistically significant *z*-scores. *z*-Scores indicate clustering, the larger the *z*-score, the higher the clustering. A high *z*-score and low *p*-value indicate a statistically significant hot spot, or a low probability that the geographic distribution is random. The negative/positive 3's indicate 99% confidence level (±2.58 *z*-score and <0.01 *p*-value), negative/positive 2's indicate 95% confidence level (±1.96 *z*-score and <0.05 *p*-value), negative/positive 1's indicate 90% confidence level (±1.65 *z*-score and <0.10 *p*-value), and finally 0's are nonsignificant points. The maximum distance input for each well to have one neighbor resulted in some features having >1,000 neighbors making our *z*-scores less reliable, so we used the average distance for eight wells in our analyses.

**FIG. 1.**
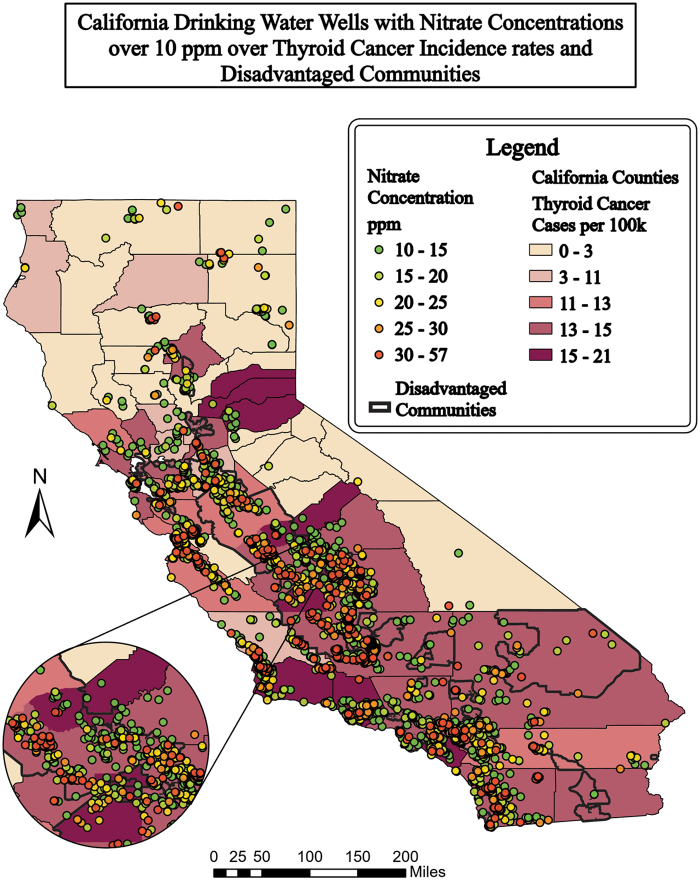
Drinking water wells concentration >10 ppm (GAMA, 2020) in California, DACs (OEHHA, 2018), and Central Valley (USGS, 2009) over thyroid incidences per 100,000 people per county (CCR, 2019). Map generated by first author. DAC, disadvantaged communities.

Thyroid cancer incidence data per 100,000 people were classified using Jenks Natural Breaks from 0 to 21 per 100,000 in ArcGIS Pro. Jenks Natural Breaks are determined statistically by variation among peaks or dips in the data. Natural breaks worked best for our study with the 0–15 cases, or “unstable counties,” which were accordingly all represented under one classification (0–3 per 100,000 in Fig. 1). Displaying thyroid cancer incidence with a colorful gradient will show the areas of California with the highest thyroid cancer rate. We then mapped well points of five incremental ranges (increasing by 5 ppm) over the thyroid cancer rate. Shape files of DACs from CalEnviroScreen and the Central Valley from USGS were added to ArcGIS Pro. We used the “spatial join” tool to count wells within California counties, DACs, and the Central Valley.

### Statistical analysis

Two different statistical tests were chosen to test our two hypotheses: (1) a correlation between nitrate well contamination and thyroid cancer, and (2) higher thyroid cancer incidence and well nitrate concentrations in DACs and the Central Valley than other areas of California. Nonparametric Spearman's Rho correlation test was utilized to determine if there was a correlation between well nitrate concentrations and thyroid cancer incidence (Spearman, 1904). Nonparametric tests were chosen due to the non-normal distribution of the data (Pallant, 2016). The nonparametric, Mann–Whitney *U*-test was used to test if the DAC and non-DAC and Central Valley and non-Central Valley thyroid rates and nitrate well concentrations were independent (Mann and Whitney, 1947). Data exported from ArcGIS Pro were used for statistical analysis (Supplementary Fig. S1) in IBM SPSS Statistic version 26 statistical software (IBM Corp., 2019).

Nitrate well contamination per county was compared with county-level thyroid cancer data by various metrics since data were not available on how many people are served by each of the individual wells. For each county, thyroid cases per 100,000 were compared with the percentage wells exceeding 5 and 10 ppm nitrate, the number of wells >5 and 10 ppm nitrate per 100,000 people and per square mile as well as hot spots per 100,000 and per square mile. Outliers outside of three standard deviations from the mean (99.7% confidence interval) were eliminated before statistical tests were conducted.

## Results

### Geospatial distribution of high nitrate concentrations

Using the “Dissolve” data management tool on ArcGIS Pro, 800,000 well nitrate concentration data points were aggregated from the GAMA database down to 43,967 well points. One hundred twenty-five points were removed as outliers (over three standard deviations from the mean nitrate concentration of wells >5 ppm, *M* = 12.6 ppm, σ = 15.1 ppm). Figure 1 display well points with concentrations >10 ppm nitrate (the U.S. federal MCL). Supplementary Figure S1 includes wells >5 ppm (half the federal MCL). Of the wells with >10 ppm average nitrate concentration, 38% are in DACs, 39% are in the Central Valley, and 27% are in DACs within the Central Valley. Of the total land area of California (155,879 mi^2^), 14% constitute DACs and 13% constitute the Central Valley. DACs comprise 47% of the Central Valley.

**FIG. 2.**
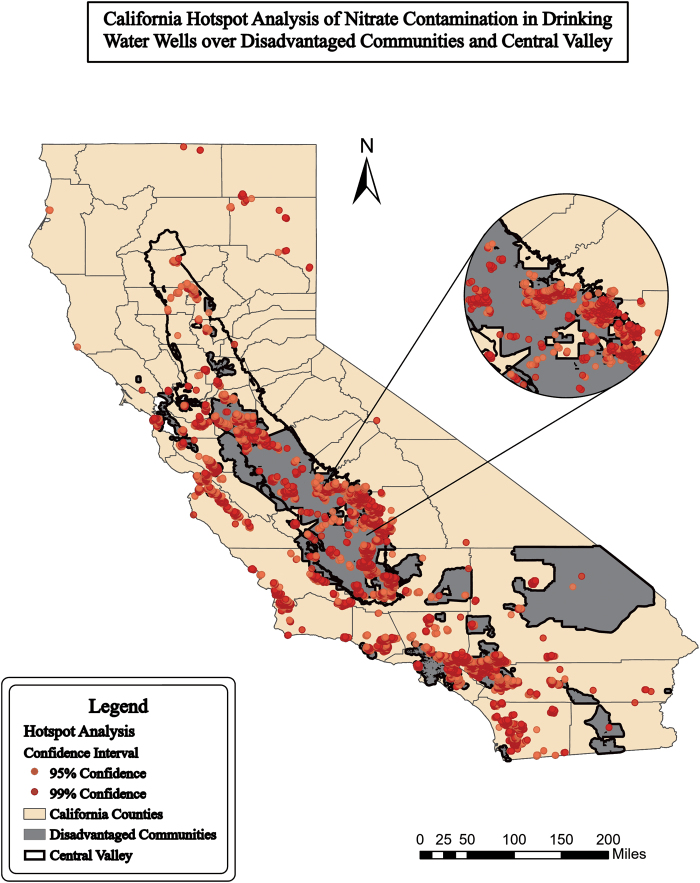
Hot spot analysis (95% confidence) of nitrate in wells in California (GAMA, 2020), DACs (OEHHA, 2018) over the Central Valley (USGS, 2009) Distance band was 9,800 ft. Map created by first author.

**FIG. 3.**
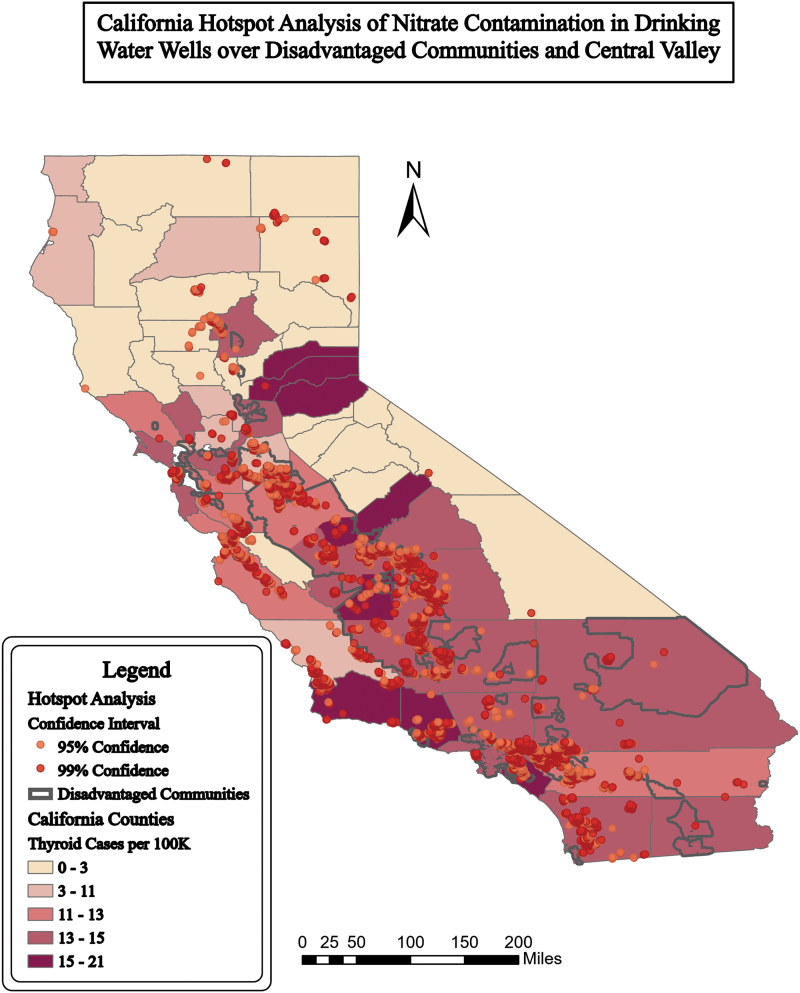
Hot spot analysis (95% confidence) of nitrate in wells in California (GAMA, 2020), DACs (OEHHA, 2018) over thyroid incidences per 100,000 people per county (CCR, 2019). Distance band was 9,800 ft. Map created by first author.

Hot spot analysis of the nitrate well point data resulted in 7,503 hot spots with >95% confidence mapped in Figs. 2 and 3. Forty-one percent of hot spots (*n* = 3,042 hot spots) were in DACs, 42% (*n* = 3,153) were in the Central Valley, and 28% (*n* = 2,124) were in DACs within the Central Valley. Hot spots were found in all counties where DACs were present, except Yuba. Supplementary Figure S2 shows the hot spot analysis of wells with a maximum of one neighbor.

### Statistical analysis—Spearman's Rho correlations

Results from Spearman's Rho correlation test and sensitivity analysis between nitrate well contamination and thyroid cancer incidences are summarized in Table 1. Five of the eight different comparisons run between wells >10 and 5 ppm and hot spots per 100,000 people, per square mile, or percentage wells and thyroid cancer incidences had statistically significant (*p* < 0.05) results. None of the nitrate variables per 100,000 people had statistically significant correlation with thyroid cancer incidence per 100,000 people. Most of the comparisons between nitrate well contamination per square mile and percentage contaminated wells and thyroid cancer incidence had statistically significant associations but had some limitations when considering the counties with 0–15 case counties.

**Table 1.** Spearman's Rho Correlation Test and Sensitivity Analysis Between Nitrate Well Contamination and Thyroid Cancer Incidences per 100,000 People

No.	Nitrate contamination	Mean	SD	0–15 case county assumption	*n*	*r*_s_	*p*
1	Wells >10 ppm per 100,000 people	13	17	0	56	0.040	0.77
				1		0.035	0.80
				7.5		0.080	0.56
				15	53	−0.17	0.22
				Excluded	36	−0.055	0.75
2	Wells >5 ppm per 100,000 people	34	37	0	58	−0.15	0.28
				1		−0.14	0.30
				7.5		−0.037	0.78
				15	56	−0.21	0.88
				Excluded	36	−0.089	0.60
3	Nitrate hot spots per 100,000 people	21	32	0	56	0.22	0.10
				1		0.23	0.091
				7.5		0.21	0.13
				15	54	−0.21	0.14
				Excluded	35	0.024	0.89
4	Wells >10 ppm per square mile	0.022	0.033	0	57	0.56	<0.001^*^
				1		0.53	<0.001^*^
				7.5		0.26	0.050
				15	55	−0.41	0.002^*^
				Excluded	36	0.066	0.70
5	Wells >5 ppm per square mile	0.059	0.086	0	57	0.56	<0.001^*^
				1		0.51	<0.001^*^
				7.5		0.22	0.096
				15	56	−0.40	0.002^*^
				Excluded	36	0.11	0.53
6	Hot spots per square mile	0.054	0.093	0	57	0.54	<0.001^*^
				1		0.51	<0.001^*^
				7.5		0.30	0.025^*^
				15	55	−0.40	0.003^*^
				Excluded	36	0.082	0.64
7	Percent wells >10 ppm	6.1	11	0	57	0.47	<0.001^*^
				1		0.45	<0.001^*^
				7.5		0.23	0.089
				15	55	−0.41	0.002^*^
				Excluded	36	−0.12	0.50
8	Percent wells >5 ppm	14	15	0	56	0.48	<0.001^*^
				1		0.44	<0.001^*^
				7.5		0.17	0.20
				15	53	−0.46	0.001^*^
				Excluded	36	−0.14	0.41

^*^ Statistically significant at the 95% confidence interval (*p* < 0.05).

SD, standard deviation.

For wells >10 ppm nitrate per square mile, the mean was 0.022 with standard deviation of 0.033. One outlier, San Francisco, was removed from the dataset before further statistical analysis. There was a statistically significant correlation between thyroid cancer incidence per 100,000 people and wells >10 ppm nitrate per square mile when 0–15 case counties were assumed to have one cancer incidence per county (*r_s_* = 0.53, *p* < 0.001, *n* = 57) and zero cancer incidence per county (*r_s_* = 0.56, *p* < 0.001, *n* = 57). A slight statistical significance was found when 0–15 case counties were assumed to have 7.5 cancer incidences per county (*r_s_* = 0.26, *p* = 0.05, *n* = 57). There was a statistically significant negative correlation coefficient (*r_s_* = −0.46) when 0–15 case counties were assumed to have 15 thyroid cancer incidences per county (*p* = 0.002, *n* = 56). When unstable counties were omitted from the dataset, there was no statistical significance between the two variables (*r_s_* = 0.066, *p* = 0.70, *n* = 36).

For wells >5 ppm nitrate per square mile, the mean was 0.059 with standard deviation of 0.086. San Francisco was removed as an outlier from the dataset before further statistical analysis. A statistically significant correlation was found between thyroid incidence per 100,000 people and wells >5 ppm nitrate per square mile when 0–15 case counties were assumed to have one cancer incidence per county (*r_s_* = 0.51, *p* < 0.001, *n* = 57) and zero cancer incidences per county (*r_s_* = 0.56, *p* < 0.001, *n* = 57). There was a slight statistical significance for when 0–15 case counties were assumed to have 7.5 cancer incidences per county when compared with wells >5 ppm per square mile (*r_s_* = 0.22, *p* = 0.096, *n* = 57). A statistically significant negative correlation coefficient (*r_s_* = −0.40) was found when 0–15 case counties were assumed to have 15 thyroid cancer incidences per county (*p* = 0.002, *n* = 56). When 0–15 case counties were omitted from the dataset, there was not a statistically significant correlation between the two variables (*r_s_* = 0.11, *p* = 0.53, *n* = 36).

For nitrate hot spots per square mile, the mean was 0.054 hot spots per square mile with standard deviation of 0.093. San Francisco was omitted as an outlier from the dataset before further statistical analysis. A statistically significant correlation was found between thyroid incidence per 100,000 people per county and hot spots per square mile when 0–15 case counties were assumed to have one cancer incidence per county (*r_s_* = 0.51, *p* < 0.001, *n* = 57), zero cancer incidence per county (*r_s_* = 0.54, *p* < 0.001, *n* = 57), and 7.5 cancer incidences per county (*r_s_* = 0.30, *p* = 0.025, *n* = 57). There was a statistically significant negative correlation coefficient (*r_s_* = −0.42) when 0–15 case counties were assumed to have 15 thyroid cancer incidences per county (*p* = 0.003, *n* = 55). When 0–15 case counties were omitted from the dataset, there was no statistically significant correlation between the two variables (*r_s_* = 0.082, *p* = 0.64, *n* = 36).

For percentage of total wells >10 ppm nitrate, the mean was 6.1% with standard deviation of 11%. A single outlier, Merced, was removed from the dataset before further statistical analysis. A statistically significant correlation was found between thyroid incidence per 100,000 people per county and percentage of wells >10 ppm nitrate when 0–15 case counties were assumed to have one cancer incidence per county (*r_s_* = 0.45, *p* < 0.001, *n* = 57) and zero cancer incidence per county (*r_s_* = 0.47, *p* < 0.001, *n* = 57). There was a slight statistically significant correlation when 0–15 case counties were assumed to have 7.5 cancer incidences per county (*r_s_* = 0.23, *p* = 0.089, *n* = 57). A statistically significant negative correlation coefficient (*r_s_* = −0.41) was found when 0–15 case counties were assumed to have 15 thyroid cancer incidences per county (*p* = 0.002, *n* = 57). When 0–15 case counties were omitted from the dataset, no statistically significant correlation was found between the two variables (*r_s_* = −0.12, *p* = 0.50, *n* = 36).

For percentage of total wells >5 ppm nitrate, the mean was 14% with standard deviation of 15%. Two outliers, Merced and San Francisco, were removed from the dataset before further statistical analysis. A statistically significant correlation was found between thyroid incidence per 100,000 people per county and percentage of wells >5 ppm nitrate when 0–15 case counties were assumed to have one cancer incidence per county (*r_s_* = 0.44, *p* < 0.001, *n* = 56) and zero cancer incidence per county (*r_s_* = 0.48, *p* < 0.001, *n* = 56). There was no statistical correlation when 0–15 case counties were assumed to have 7.5 cancer incidences per county (*r_s_* = 0.17, *p* = 0.20, *n* = 56). A statistically significant negative correlation coefficient (*r_s_* = −0.46) was found when 0–15 case counties were assumed to have 15 thyroid cancer incidences per county (*p* < 0.001, *n* = 56). When unstable counties were omitted from the dataset, there was no statistically significant correlation between the two variables (*r_s_* = −0.14, *p* = 0.41, *n* = 35).

### Population statistics

Nineteen counties have >200 mi^2^ within the Central Valley shape file. However, Contra Costa county (221 mi^2^ area in the valley) was excluded since it is considered to be in the San Francisco Bay Area rather than the Central Valley (EDD, 2020). Twenty-four percent of California's population are in DAC, and 19% of California's population are in Central Valley counties. In California, DACs have a higher population density (428 persons/mi^2^) than non-DACs (218 people/mi^2^). The Central Valley also had a higher population density of 362 people/mi^2^ versus 230 people/mi^2^ in the non-Central Valley. All of California has a population density of 247 people/mi^2^ based on 2014 data.

In DACs, there are 1.4 wells per 1,000 people compared with 1.0 wells per 1,000 people in non-DACs and 1.1 wells per 1,000 in all of California. In the Central Valley, there are 2.1 wells per 1,000 people and the non-Central Valley has 0.9 wells per 1,000 people. Twenty-nine percent of all wells are in DACs and 35% of wells are in the Central Valley.

### Thyroid cancer incidence

Throughout the 58 counties in California, thyroid cancer cases ranged from 1 to 1,482 per county. Thyroid cancer rates ranged from 0 to 21 per 100,000 when unstable counties were considered to have one thyroid cancer case. Overall, California has an average thyroid cancer incidence of 9.1 per 100,000 people. In counties with DACs, the average was 12.6 thyroid cancer cases per 100,000, and in those without DACs (non-DACs) it was 6.02 thyroid cancer cases per 100,000 people when 0–15 case counties were assumed to have one thyroid cancer case. The differences in thyroid cancer cases per 100,000 people were statistically significant between DACs and non-DACs (*U* = 666.5, *p* ≤ 0.001, *n* = 58).

In the Central Valley, there is an average of 9.6 thyroid cancer cases per 100,000, and outside the Central Valley it was 8.9 per 100,000 people when 0–15 case counties were assumed to have one thyroid cancer incidence. The difference in thyroid cancer cases per 100,000 between the Central Valley and non-Central Valley counties was not statistically significant (*U* = 371.5, *p* = 0.99, *n* = 58).

## Discussion

High amounts of nitrate well contamination predominantly exist in DACs. Forty-one percent of total hot spots are in DACs when DACs make up 14% of California's total land area and 24% of the total population. These communities are disproportionately affected by contaminated drinking water wells compared with the rest of California. Balazs *et al.* (2011) also found higher nitrate well contamination in low-income and Hispanic communities in the San Joaquin Valley. With higher population density in DACs and the amount of people per well, a larger number of people are exposed to wells with high levels of nitrate. High nitrate concentrations can pose health problems.

Previously, much of the health focus related to elevated nitrate levels in drinking water has been on methemoglobinemia, but other more chronic conditions such as thyroid cancer may be linked to higher ingestion of nitrates from contaminated drinking water (Comly, 1987; Ward *et al.*, 2010; Drozd *et al.*, 2016; Njeze *et al.*, 2014). Thyroid cancer incidences per 100,000 people in DACs were over two times higher in counties with DACs compared with counties without DACs (Supplementary Fig. S3). Similar to the Vermont study, the hypothesis in this study was that high thyroid cancer incidence rates are related to environmental and socioeconomic factors (Hanley *et al.*, 2015). Environmental pollutants and socioeconomic factors contribute to the designation of DACs in California (OEHHA, 2018).

Rural and urban areas are both affected by nitrate contamination. Rural areas in California are known to have sensitive populations who may be unfairly burdened by pollution (Balazs *et al.*, 2011). Figures 1 and 2 show that rural agricultural areas, much of the central part of California, held a majority of the high nitrate concentrations over EPA standards of 10 ppm (Figs. 1 and 2). In urban areas, fertilizers from lawns, wastewater leakage, or atmospheric depositions also contribute to the nitrate contamination. While agriculture is the main focus for mitigation, urban areas have high concentrations of nitrate contamination in smaller areas (Wakida and Lerner, 2005).

Our analysis resulted in statistically significant correlations between nitrate well contamination per square mile and percentage of wells >10 and 5 ppm and thyroid cancer incidences per 100,000 people. However, a statistically significant correlation was not found between contaminated wells per 100,000 and thyroid cancer rates. This could potentially be due to residents drinking bottled water (Fernandez-Bou *et al.*, 2020; Rosinger and Young, 2020), or from municipal and household water treatment.

Particularly after the Flint water crisis, Americans questioned their water sources and the safety of their tap water. A 2020 study investigated tap water avoidance, and found an increase in avoidance in children (ages 2–19) from 12% avoiding tap water in 2013–2014 to 16% in 2015–2016, which corresponds to timing of the Flint water crisis. Hispanic and Black children, low-income, and low education families were twice more likely to drink bottled water compared with White children (Rosinger and Young, 2020). Furthermore, nitrate contamination per square mile may be more appropriate than wells per 100,000 people in more populous areas where deep municipal wells serve large amounts of the population.

More granular data of thyroid cancer incidence and thyroid disorders are needed by census tract for future analyses. The unstable or 0–15 case county sensitivity analysis demonstrated the limitations of open thyroid cancer incidence data for California. In most cases, when 0–15 thyroid case counties were assumed to have 15 cases, there was a statistically significant negative correlation, but most counties probably have much <15 cases and 7.5 and even one per 100,000 are more valid assumptions.

This study provides a methodology for similar preliminary analysis in other states and countries with open data before pursuing Institutional Review Board (IRB) approval for more specific data. With more localized data, multivariable regression is recommended to control for other confounding variables for thyroid cancer such as population, socioeconomic factors, and other underlying factors for thyroid cancer such as radiation, obesity, and high or low iodine in the diet (Drozd *et al.*, 2016; ACS, 2019).

Even if data were available by census tract, there may be limitations since people living in DACs do not have the same access to health care as people in non-DACs (OEHHA, 2018). Lack of health care access can cause under-reporting of medical issues. In addition to medical data, more qualitative research and interviews with random sampling are needed in these communities related to their health, other environmental exposures, and bottled water consumption. Unmonitored private wells in the rural parts of California could also be contaminated and cause health risk for those who drink water from them. A better well monitoring system is needed for the health of the residents and a more robust analysis of health impacts from contaminants (Jensen *et al.*, 2012; Harter *et al.*, 2012).

Water treatment and source reduction are two major parallel solutions for nitrate contamination in drinking water wells. No single policy will be the answer. People who get their drinking water from wells contaminated with nitrates need to either get their drinking water from another source (uncontaminated ground or surface water) or use proper treatment technologies such as ion modified granulated activated carbon, reverse-osmosis systems, and biological denitrification (Jensen *et al.*, 2012). Changes in nitrate loading will take decades to reduce the amount of nitrate present in groundwater (Pennino *et al.*, 2017). For example, a study in Nebraska found decreases of nitrate contamination in two of their management areas under irrigated cropland after over 20 years of fertilizer restrictions that were rare among the other management areas (Exner *et al.*, 2014).

State and federal governments and agencies and the general public (farmers and consumers) should continue to promote, scale up, demand, and incentivize practices that reduce nitrate loading in agriculture such as crop rotation, timing, and rate of fertilizer application, and efficient irrigation systems (Ribaudo *et al.*, 2011; Davidson *et al.*, 2012; Harter *et al.*, 2012). Education programs for farmers about mitigation techniques to decrease nitrate contamination are also important (Ribaudo *et al.*, 2011). Furthermore, “information on how to conduct and interpret nitrogen tests and how to successfully implement new practices can reduce the overall costs and increase adoption rates” according to a USDA study (Ribaudo *et al.*, 2011).

Based on this study's preliminary geospatial and statistical analyses of nitrate well contamination and thyroid cancer, future research in several areas is recommended. First, adding surface water data would be beneficial. Although surface water usually has better water quality than groundwater (Schaider *et al.*, 2019), there could still be areas of contamination, particularly with agricultural intensification and urbanization and associated runoff.

Future research is needed on the effects of nitrate contamination on other thyroid dysfunctions, including goiter, hyperthyroidism, or hypothyroidism and synergistic effects with other contaminants such as pesticides (Ward *et al.*, 2010; Ward *et al.*, 2018). Nitrates compete with the uptake of iodine, and can affect the thyroid gland and TSH levels causing goiter, an abnormal enlargement of the thyroid gland (Gatseva and Argirova, 2008; Ward *et al.*, 2018). Consumption of nitrate-contaminated drinking water is also associated with other cancers (e.g., bladder, colorectal, esophagus, stomach cancer, etc.) (Njeze *et al.*, 2014; Ward *et al.*, 2018), and should be geospatially and statistically analyzed in California and other areas.

## Summary

In summary, high nitrate contamination in drinking water wells associated with thyroid cancer is prevalent in DACs and the Central Valley in California. Approximately 40% of nitrate hot spots were found in the Central Valley and in DACs where cropland can account for up to 96% of the nitrate loading (Harter *et al.*, 2012). Geospatial analysis shows a high amount of wells predominantly in the Central Valley of California with intense agriculture, thus residents are often exposed to drinking water contaminated with nitrates. DACs are also in urban areas, with other sources of contamination including wastewater leakages or fertilizers on lawns (Wakida and Lerner, 2005; Cadenasso *et al.*, 2008). However, some communities in DACs may rely on bottled water where there is distrust in their water source (Fernandez-Bou *et al.*, 2020; Rosinger and Young, 2020). DACs contained two times greater thyroid cancer incidence compared with non-DACs.

Statistically significant correlations were found between thyroid cancer rates and nitrate-contaminated wells per square mile and percentage of total wells but not nitrate-contaminated wells per 100,000 people. Other studies indicate correlation between thyroid cancer and nitrate well contamination (Ward *et al.*, 2010; Drozd *et al.*, 2016). This study provides methods for other states and countries to analyze links between thyroid and other cancers to nitrate contamination in drinking water wells with open data.

For further study, census tract or more localized data of thyroid cancer incidences are needed to better analyze regions and DACs in California that are disproportionately affected by environmental factors. In addition, other thyroid disorders and cancers (bladder, colorectal, esophagus, stomach, etc.) associated with nitrates should be incorporated into future research.

Thyroid cancer is disproportionately affecting women, and incidence rates are increasing and pervasive in DACs linked to probable environmental exposures (Horn-Ross *et al.*, 2014; Hanley *et al.*, 2015). Funding and policies are needed to reduce nitrate loading and treat contaminated water. By partnering with organizations committed to grassroots activities to champion environmental improvements in DACs like the Community Water Center in California (CWC, 2021), the data and approach used in this study can be leveraged to conduct further research, and advocate for drinking water treatment and nitrate reduction funding to ensure environmental justice (see Supplementary Data Fact Sheet).

## Supplementary Material

Supplemental data

Supplemental data

Supplemental data

Supplemental data

Supplemental data

## Supplementary Material

Supplementary Figure S3

Supplementary Data Fact Sheet
