# Supplementary material for: Water, Health, and Environmental Justice in California: Geospatial Analysis of Nitrate Contamination and Thyroid Cancer
Source: Environ Eng Sci. 2021 May 24;38(5):377–88. doi: 10.1089/ees.2020.0315 (PMC8165459; doi:10.1089/ees.2020.0315)
Supplement: Supplemental data [file Supp_TableS1.docx]

**Supplementary Table 1.** Workflow of geospatial and statistical analysis of nitrate well and thyroid cancer data in California.

| **COLLECTION** | **GEOSPATIAL ANALYSIS** | **EXCEL** | **STATISTICAL ANALYSIS** |
| --- | --- | --- | --- |
| ***Well Points and Nitrate concentrations***  Waterboards GAMA website and export all to Excel (over 800,000 points)  *Calculated area:* ‘calculate geometry’ tool in ArcGIS Pro  *Thyroid Cancer incidence rate:*  (Cases/population)* 100,000  ***Thyroid Cancer Incidence***  California Cancer Registry:  thyroid cancer cases per county from the year 2014.  California State Association of Counties: Obtain population by county | ***Aggregate Data***  Using ‘dissolve’ tool aggregate well points  Select out outliers  ***Hotspot Analysis***  ‘Calculate Nearest Neighbor’ ~  *Input:* well points from last step  *Neighbors:* 1 and 8  (2 separate analyses) | ***Export to Excel:***  *‘Table to excel’ tool:* export to an Excel file.  *Separate by county:*  use ‘COUNTIF’ function to find amount of each well point in each county  ***Select Well Points***  Select well points with nitrate concentration over 5 and 10 ppm and hotspots with greater than 95% confidence from ArcGIS Pro | ***Non-parametric, Mann-Whitney U-Test:***  Used to test DAC and non-DAC and Central Valley and non-Central Valley thyroid rates independence.  ***Import to IBM SPSS Statistics version 26***  *Non-parametric Spearman Rho correlation test:* outliers were eliminated.  For correlation between well nitrate concentrations and thyroid cancer incidence |
| ***Land Area Shapefiles obtained***  *CalEPA-CalEnviroScreen 3.0:*  Disadvantaged Communities  *California Open Data Portal:* CA Counties  *United States Geographical Survey (USGS):* Central Valley | ***Getis-Ord GI****  *Input class:* well points from last step.    *Input field:* mean nitrate concentration  *Conceptualization of spatial relationship:* fixed distance  *Distance Band:* output from ‘Calculate Nearest Neighbor’ | Completed Excel with all necessary data |  |
