# Supplementary material for: Water, Health, and Environmental Justice in California: Geospatial Analysis of Nitrate Contamination and Thyroid Cancer
Source: Environ Eng Sci. 2021 May 24;38(5):377–88. doi: 10.1089/ees.2020.0315 (PMC8165459; doi:10.1089/ees.2020.0315)
Supplement: Supplemental data [file Supp_FigS1.docx]

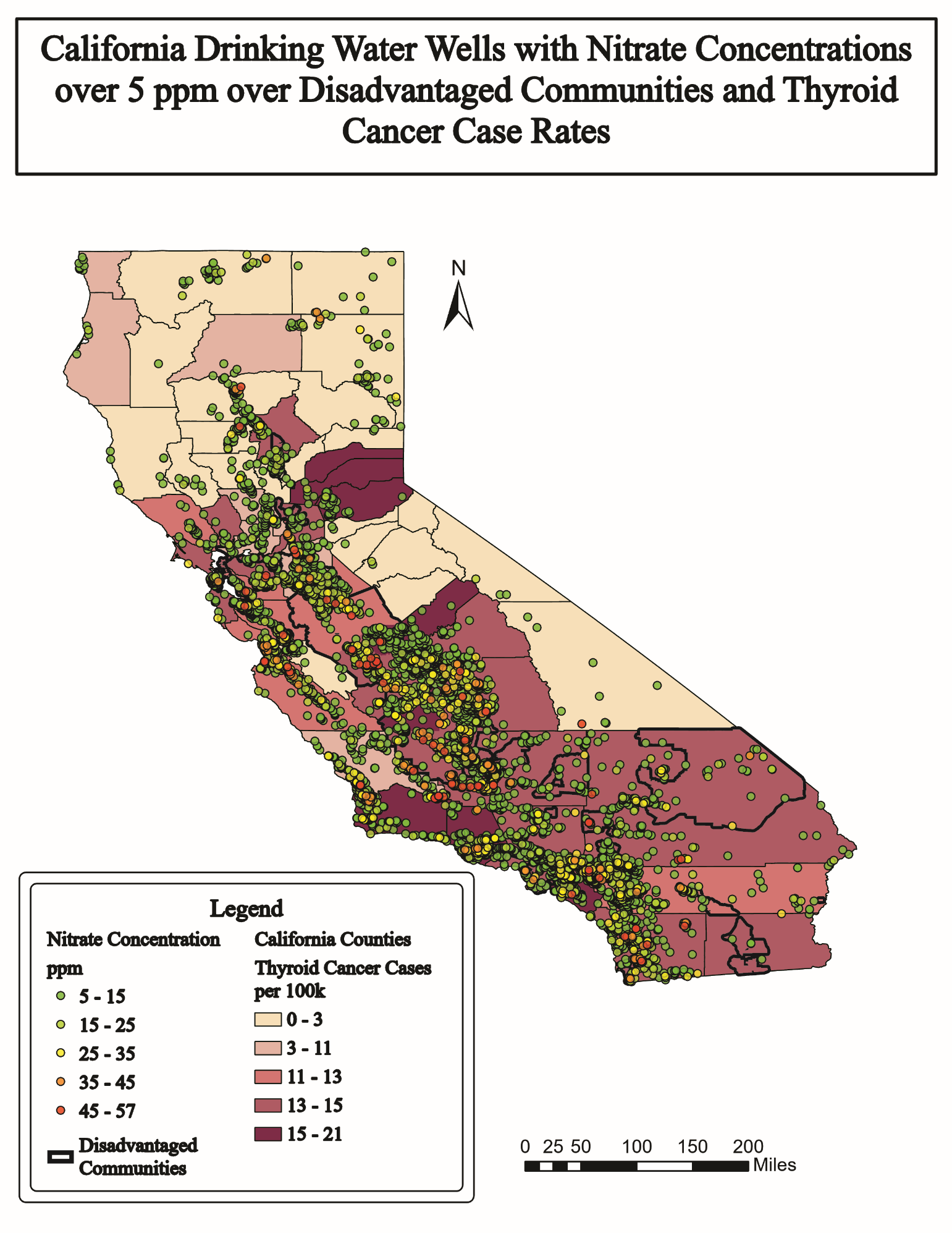


Supplementary Figure 1. Drinking water wells concentration over 5 ppm (GAMA, 2020) in California, disadvantaged communities (OEHHA, 2018), over thyroid incidence per 100,000 people per county (CCR, 2019). Map created by first author.
