# Supplementary material for: Water, Health, and Environmental Justice in California: Geospatial Analysis of Nitrate Contamination and Thyroid Cancer
Source: Environ Eng Sci. 2021 May 24;38(5):377–88. doi: 10.1089/ees.2020.0315 (PMC8165459; doi:10.1089/ees.2020.0315)
Supplement: Supplemental data [file Supp_FigS2.docx]

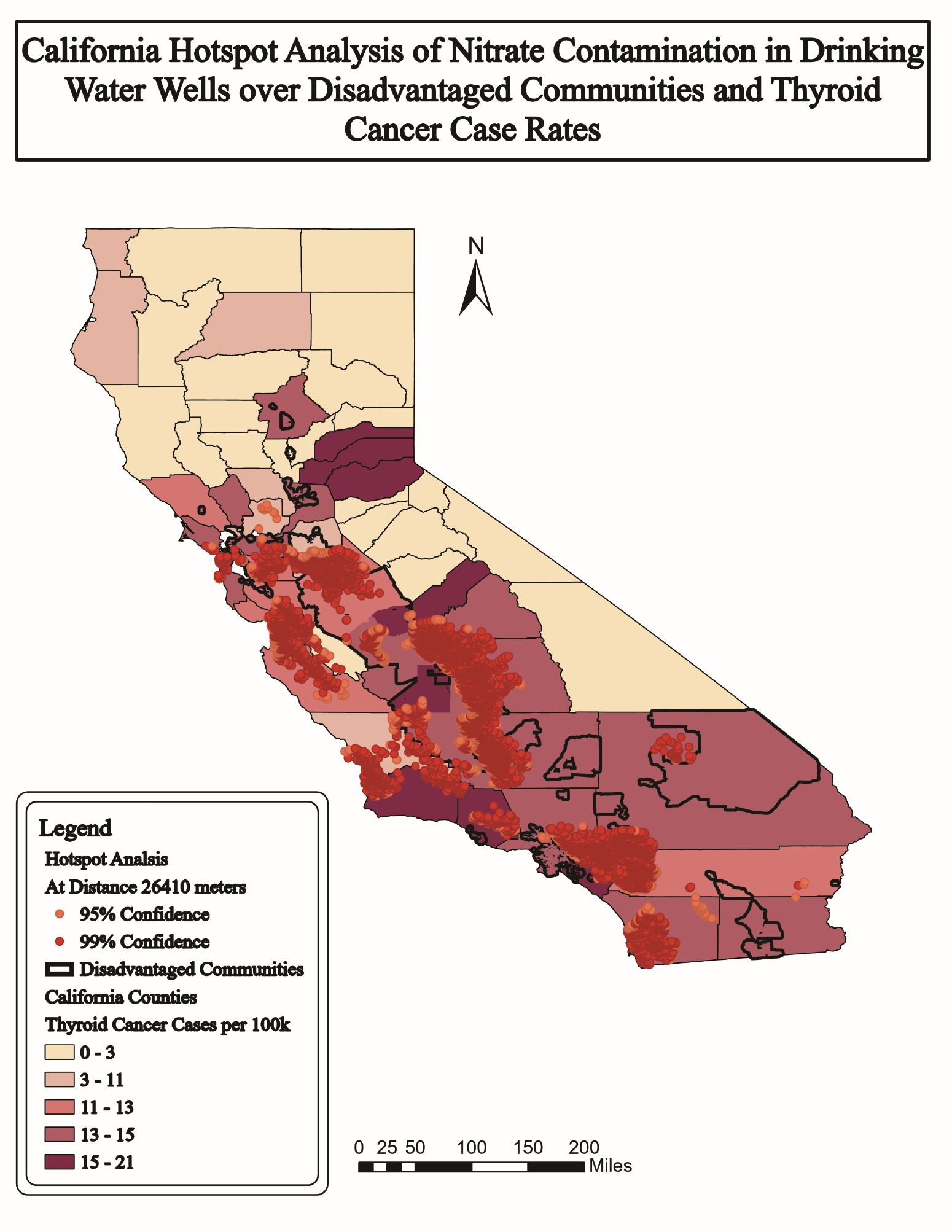


Supplementary Figure 2. Hotspot analysis (95% confidence) of nitrate in wells in California (GAMA, 2020), disadvantaged communities (OEHHA, 2018), over thyroid incidence per 100,000 people per county (CCR, 2019). Distance band was 26,410 meters. Map created by first author.
