# Supplementary material for: Water, Health, and Environmental Justice in California: Geospatial Analysis of Nitrate Contamination and Thyroid Cancer
Source: Environ Eng Sci. 2021 May 24;38(5):377–88. doi: 10.1089/ees.2020.0315 (PMC8165459; doi:10.1089/ees.2020.0315)
Supplement: Supplemental data [file Supp_DataS1.docx]

**
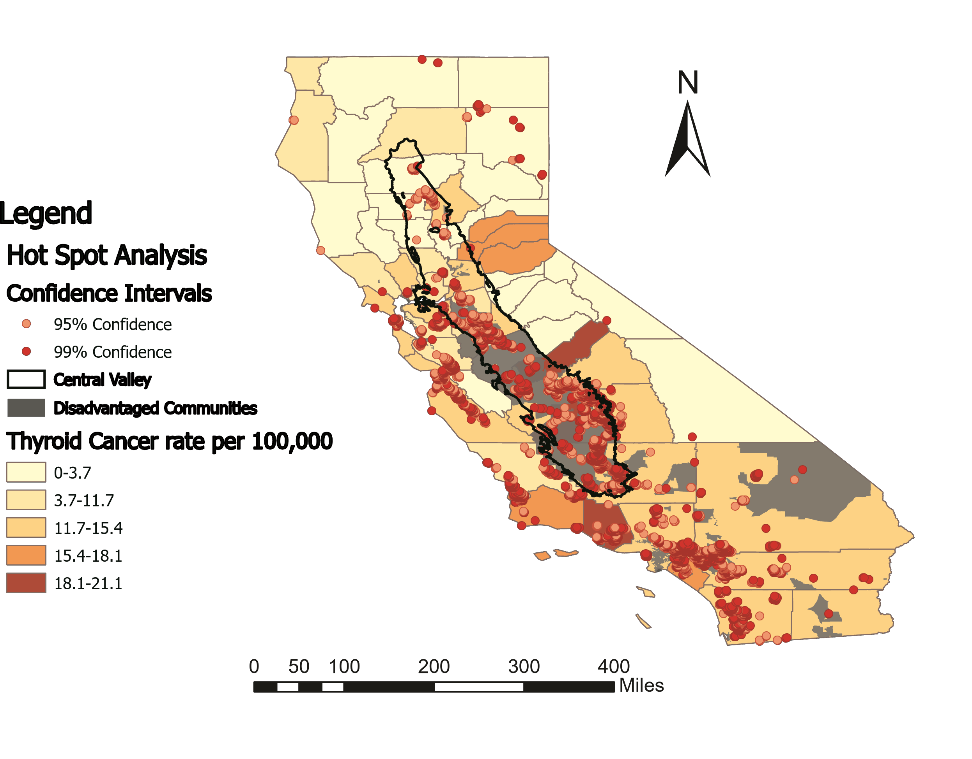
**
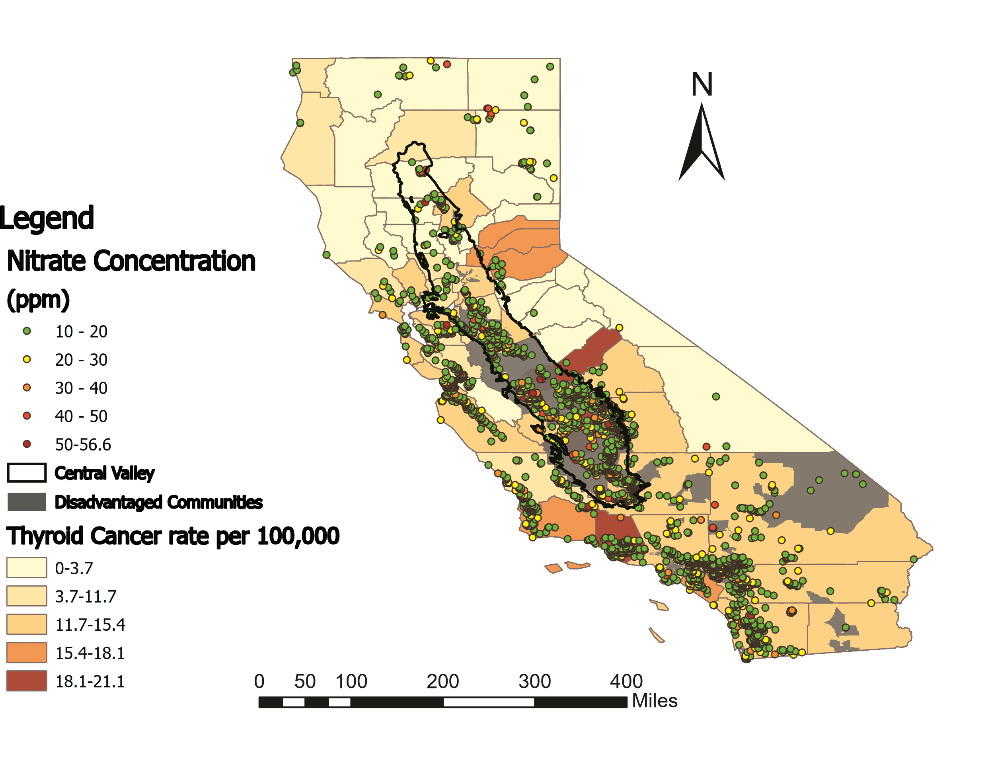


- In California, thyroid cancer incidence is 12.4 per 100,000 people. In women incidence rates superseded men at 19.4 per 100,000 people and 6.9 per 100,000 respectively (based off 2017 data). These rates are increasing at a 4.6 and 4.1 average annual percentage rate (based of 2005 – 2014 data)
- Nitrates are a common anthropogenic pollutant. Nitrates come from agriculture, industry, and wastewater sources. In California, the Central Valley produces one-fourth of the United States food supply. Disadvantaged communities (DACs) are disproportionality affected by environmental health hazards.
- 40% of wells with nitrate contamination over 10 ppm (the federal MCL) in the Central Valley and 41% of hotspots. In DACs 38% of wells over 10 ppm were found and 42% of Hotspots.
- Studies recently have focused on the relationship between the increase in nitrates and thyroid cancer. In California, we found statistically significant (p <0.05) correlation between high nitrate contaminations per square mile and thyroid cancer incidence. DACs had two times greater the rate of thyroid cancer incidence compared to non-DACs.

**Water, Health, and Environmental Justice in California: Geospatial Analysis of Nitrate Contamination and Thyroid Cancer Fact Sheet**
